# Point-of-Care Ultrasound by Nonexpert Operators Demonstrates High Sensitivity and Specificity in Detecting Gallstones: Data from the Samoa Typhoid Fever Control Program

**DOI:** 10.4269/ajtmh.21-0973

**Published:** 2022-01-10

**Authors:** Seth A. Hoffman, Sachin N. Desai, Michael J. Sikorski, Glenn Fatupaito, Siaosi Tupua, Robert E. Thomsen, Savitra Rambocus, Susana Nimarota-Brown, Linatupu L. Punimata, Michelle Sialeipata, Chandler F. Tuilagi, Jane Han, Roy M. Robins-Browne, Take K. Naseri, Myron M. Levine

**Affiliations:** ^1^ Center for Vaccine Development and Global Health, University of Maryland School of Medicine, Baltimore, Maryland;; ^2^ Department of Medicine, University of Maryland School of Medicine, Baltimore, Maryland;; ^3^ Department of Pediatrics, University of Maryland School of Medicine, Baltimore, Maryland;; ^4^ Samoa Typhoid Fever Control Program, Ministry of Health, Government of Samoa, Apia, Samoa;; ^5^ Institute for Genome Sciences, University of Maryland School of Medicine, Baltimore, Maryland;; ^6^ Department of Microbiology and Immunology, University of Maryland School of Medicine, Baltimore, Maryland;; ^7^ Tupua Tamasese Meaole Hospital, Ministry of Health, Government of Samoa, Apia, Samoa;; ^8^ Microbiological Diagnostic Unit Public Health Laboratory, Department of Microbiology and Immunology, The University of Melbourne at the Peter Doherty Institute for Infection and Immunity, Melbourne, Australia;; ^9^ Murdoch Children’s Research Institute, Royal Children’s Hospital, Melbourne, Australia

## Abstract

Approximately 90% of chronic typhoid carriers with persistent *Salmonella enterica* serovar Typhi (*S.* Typhi) gallbladder infection have gallstones. In Samoa, where typhoid fever has been endemic for many decades, risk factors predisposing to the development of gallstones are increasing among adults. The Samoa Typhoid Fever Control Program dispatches a “Typhoid Epidemiologic SWAT Team” to perform a household investigation of every blood culture-confirmed case of acute typhoid fever. Investigations include screening household contacts to detect chronic carriers. Following limited training, two nonexpert ultrasound operators performed point-of-care ultrasound (POCUS) on 120 Samoan adults from August to September 2019 to explore the feasibility of POCUS to detect individuals with gallstones during household investigations and community screenings. POCUS scans from 120 Samoan adults in three cohorts (28 food handlers, two typhoid cases and their 18 household contacts, and 72 attendees at an ambulatory clinic) were reviewed by a board-certified radiologist who deemed 96/120 scans (80%) to be interpretable. Compared with the radiologist (gold standard), the nonexpert operators successfully detected 6/7 Samoans with gallstones (85.7% sensitivity) and correctly identified 85/89 without gallstones (95.5% specificity). The proportion (24/120) of uninterpretable scans from this pilot that used minimally trained clinicians (who are neither radiologists nor ultrasound technicians) indicates the need for additional training of POCUS operators. Nevertheless, this pilot feasibility study engenders optimism that in the Samoan setting nonexperts can be trained to use POCUS to diagnose cholelithiasis, thereby helping (along with stool cultures and Vi serology) to identify possible chronic *S.* Typhi carriers.

## INTRODUCTION

Typhoid fever is endemic in the island nation of Samoa, in Oceania. Approximately 2–4% of untreated acute *Salmonella enterica* subspecies *enterica* serovar Typhi (*S.* Typhi) infections, clinical or subclinical, become “chronic carriers” who excrete for more than 12 months, and typically for decades.^1^^,^^2^ Moreover, 80–90% of known chronic carriers have gallstones and harbor *S.* Typhi in their gallbladder.^3^^,^^4^

In addition to systemic improvements to water and sanitation infrastructure, a new tool to help control endemic typhoid fever is Vi conjugate vaccine. A single dose of Typbar-TCV^®^, the first prequalified Vi conjugate, was 79–95% effective in preventing typhoid fever following immunization of infants, children, and young adults.^5^^–^^10^ However, even if high vaccination coverage substantially reduces the incidence of typhoid,^5^^–^^10^ for some years chronic *S.* Typhi carriers will remain within the population constituting a reservoir of infection that can be transmitted. Ideally, chronic carriers can be systematically identified (by stool culture and IgG Vi antibody titer) and treated if the antimicrobial susceptibility of the endemic strain(s) allows.^11^^–^^13^ Of note, in adults recently vaccinated with Vi-conjugate vaccine, Vi serology is unlikely to be useful in screening for chronic carriers.

Samoa has initiated a Typhoid Fever Control Program with: 1) a Preparatory Phase based on enhanced epidemiologic surveillance and strengthening of microbiology infrastructure to elucidate the disease burden by person, place, and time, 2) an Attack Phase that includes a mass vaccination with Typbar-TCV targeting all Samoans 1–45 years of age and introduction of Typbar-TCV into the Expanded Program on Immunization as a routine vaccine for infants co-administered with measles/mumps/rubella (MMR) vaccine, and 3) a future Consolidation Phase with intensive searches to identify and treat chronic carriers.^14^

Changes in diet and lifestyle in Samoa have led to rises in noncommunicable diseases, including obesity and metabolic syndrome,^15^^–^^22^ known risk factors for development of gallstones.^23^^,^^24^ This could impact typhoid fever control if the prevalence of chronic carriers were to increase commensurate with a rise in the prevalence of gallstones.^3^^,^^4^^,^^25^^–^^29^ The Samoa Typhoid Fever Control Program is exploring strategies to detect, cure, and monitor chronic carriers.^3^^,^^4^^,^^11^^,^^12^^,^^25^^–^^29^ Typhoid Epidemiologic SWAT Teams visit the households (or school or workplace, as relevant) of every blood culture-confirmed typhoid case to perform an epidemiologic investigation that includes the collection of three stool cultures from every household contact to identify individuals who may be asymptomatically excreting *S*. Typhi. Two indicators that increase the likelihood that an excretor is a true chronic *S*. Typhi carrier are the presence of gallstones,^4^ and a highly elevated serum IgG Vi antibody titer (found in 85–90% of chronic carriers but uncommonly in the general population).^12^^,^^25^^,^^27^^,^^28^

Recognizing that 80–90% of chronic *S.* Typhi carriers have cholelithiasis,^3^^,^^4^ we assessed the feasibility of a practical, noninvasive, field ultrasound method to identify gallstones in Samoan adults during the course of Typhoid Epidemiologic SWAT Team investigations of households of acute typhoid cases, and for future population-based surveys among older Samoan adults. The former will help identify carriers who may have transmitted *S.* Typhi to a household member, whereas the population-based surveys will help identify individuals with cholelithiasis who will become targets for additional studies (multiple stool cultures and Vi serology) to identify silent chronic carriers in the general population and certain subpopulations (e.g., individuals used as food handlers). Identifying individuals with cholelithiasis may have enhanced importance as a greater proportion of Samoan adults become vaccinated with Typbar-TCV, after which Vi serology will have reduced utility for screening.

Ultrasound screening of the right upper quadrant of the abdomen is sensitive and specific for identifying gallstones,^30^ including when performed with point-of-care ultrasound (POCUS) devices, a methodology particularly attractive for low- and middle-income country (LMIC)-settings.^31^ Conventional ultrasound machines require multiple probes to image different regions of the body. We chose the Butterfly iQ^®^ portable ultrasound probe because of its “all-in-one” design, compatibility with Apple and Android tablets/phones, and lower cost than conventional portable units.

Comparisons between nonexpert and expert POCUS operators to diagnose cholelithiasis have reported a high diagnostic accuracy, even when performed by nonexpert operators, with a sensitivity of 93% and specificity of 88%.^32^ In our pilot study of the feasibility of utilizing POCUS to detect adults with gallstones in Samoa, two physicians who were nonexperts in performing and interpreting abdominal ultrasound received minimal POCUS training, before screening Samoan adults for cholelithiasis.

## MATERIALS AND METHODS

### Ultrasound.

Typhoid Epidemiologic SWAT Team investigations of typhoid case households must be performed as soon as possible after confirmation of a case. Because many Samoans live in rural isolated villages or households that cannot be readily accessed by vehicle during rainy months and must be reached on foot, a lightweight, compact, highly portable ultrasound device was selected for evaluation. This POCUS probe (Butterfly iQ, Butterfly Network, Inc., manufactured 2019) and its proprietary imaging software for use on the Apple iPad (iPad 9.7-inch 6th Gen 128GB model A1954) were used to screen subjects for gallstones in both the short-axis/out-of-plane view (wherein the image is perpendicular to the course of the gallbladder) and the long-axis/in-plane view (the image is parallel to the course of the gallbladder), and with the subject in supine and left lateral decubitus positions (Figure 2). Images and videos were saved to Butterfly Network’s privacy-compliant server for later review (Figure 3).

### Nonexpert POCUS operators.

The nonexpert clinicians who received brief training in POCUS were a third-year U.S. internal medicine resident (S.A.H., who participated in a general clinical ultrasound workshop at the 2014 American Society of Tropical Medicine and Hygiene Annual Meeting), and a pediatric infectious diseases specialist (S.N.D.). Each nonexpert received approximately 2 hours of practical hands-on training of ultrasonography of the right-upper quadrant of the abdomen. Using both traditional and handheld POCUS devices under supervision, the nonexperts were instructed how to identify and characterize the gross morphology of the gallbladder, detect gallbladder wall thickening, and determine the contents of the gallbladder such as gallstones or biliary sludge. These skills were practiced on subjects in the supine and left lateral decubitus positions.

### Cohorts screened with POCUS by the nonexperts.

Three cohorts of indigenous Samoans were screened for gallstones by the two nonexpert clinicians using POCUS. The clinicians took turns performing POCUS scans on alternating patients. They thereupon jointly evaluated their scans (including saved image stills and video) for the presence or absence of gallstones, and recorded their consensus diagnoses.

#### Cohort 1.

(*N* = 28) was a convenience sample from 200 Samoans who had been screened as potential food handlers for employment during The Pacific Games, a sporting competition among the Pacific Island nations held every 4 years and hosted in Apia, Samoa, from July 7 to 20, 2019. The Ministry of Health considered it important that these individuals be carefully screened to detect possible silent typhoid carriers among them, lest such an individual inadvertently spread *S.* Typhi to visiting team members or visitors during the Games. Accordingly, the Samoa Ministry of Health required that each individual seeking employment as a food handler for the games be screened by having three stools or rectal swabs shown to be negative for *S*. Typhi and a serum IgG Vi antibody test that did not show a high titer—all food handlers tested negative. A convenience sample comprising 28 accessible screened food handlers underwent POCUS several weeks after completion of the Pacific Games.

#### Cohort 2.

(*N* = 20) comprised two adults with blood culture-confirmed typhoid fever diagnosed during August 2019, and 18 of their household contacts.

#### Cohort 3.

(*N* = 72) comprised healthy, asymptomatic individuals > 20 years of age, who volunteered to have POCUS of the gallbladder performed at one of four outpatient clinics (three on Upolu and one on Savaii). In these outpatient clinics, a flyer (in the Samoan language) was posted at the check-in counter 3 days in advance to alert outpatients and their accompanying family members and companions of the impending POCUS screening days.

Prior to initiation of POCUS, participating individuals signed an informed consent translated into Samoan and read to the volunteer by a Samoan member of the team. The study protocol was reviewed and deemed exempt by the University of Maryland Baltimore (UMB) Institutional Review Board (IRB: HP-00087816), “UMB IRB reviewed the information provided and has determined the submission does not require IRB review. This determination has been made with the understanding that the proposed project does not involve a systematic investigation designed to develop or contribute to generalizable knowledge OR a human participant.” Subjects were informed that although they may not have evidence of a gallstone on POCUS, they may subsequently develop gallbladder illness at a later point in life. Thus, the exam performed in this screening exercise should not take the place of a medical doctor’s formal evaluation of any abdominal issues, should such arise.

### Expert adjudication by a radiologist of the POCUS scans/videos obtained by the nonexperts.

All 120 scans were independently reviewed by a board-certified radiologist (G.F.) at Tupua Tamasese Meaole Hospital in Samoa. The radiologist was provided the login to the online Butterfly Network account associated with the Samoa Typhoid Fever Control Program to access the uploaded scans for review. Each scan was de-identified in terms of patient information and results (i.e., initial interpretations by the nonexpert operators). First, the radiologist evaluated if the scan was technically suitable to be interpreted for the presence of gallstones. The radiologist thereupon scored each technically suitable POCUS scan as positive or negative for gallstones. After the radiologist’s interpretations of all the technically suitable POCUS scans were recorded, they were compared with those of the nonexperts. The sensitivity, specificity, positive predictive value, and negative predictive value of nonexpert POCUS results were calculated using the radiologist’s diagnosis as the gold standard for the presence/absence of gallstones.

### Testing POCUS subjects with cholelithiasis for *S.* Typhi infection.

When cholelithiasis was identified in an asymptomatic individual who had not previously undergone screening for *S*. Typhi carriage, the individual was offered fecal cultures to detect *S*. Typhi. Had any participants been culture-positive, they would have been offered a 28-day course of ciprofloxacin and monitored for “cure” of carriage by a Typhoid Epidemiologic SWAT team.^33^ The monitoring would involve collection of fecal specimens every 3–6 months until 12 months after the first positive culture. If the individual persisted in excreting *S*. Typhi even after attempted eradication with a 4-week course of ciprofloxacin, monitoring would continue with additional cultures circa every 6 months.

### Statistical analysis.

Confidence intervals (CIs) were calculated using MedCalc Software Ltd. diagnostic test evaluation calculator (https://www.medcalc.org/calc/diagnostic_test.php; Version 20.009). “Exact” Clopper-Pearson confidence intervals based on the exact binomial distribution.^34^

## RESULTS

**Figure 1.**
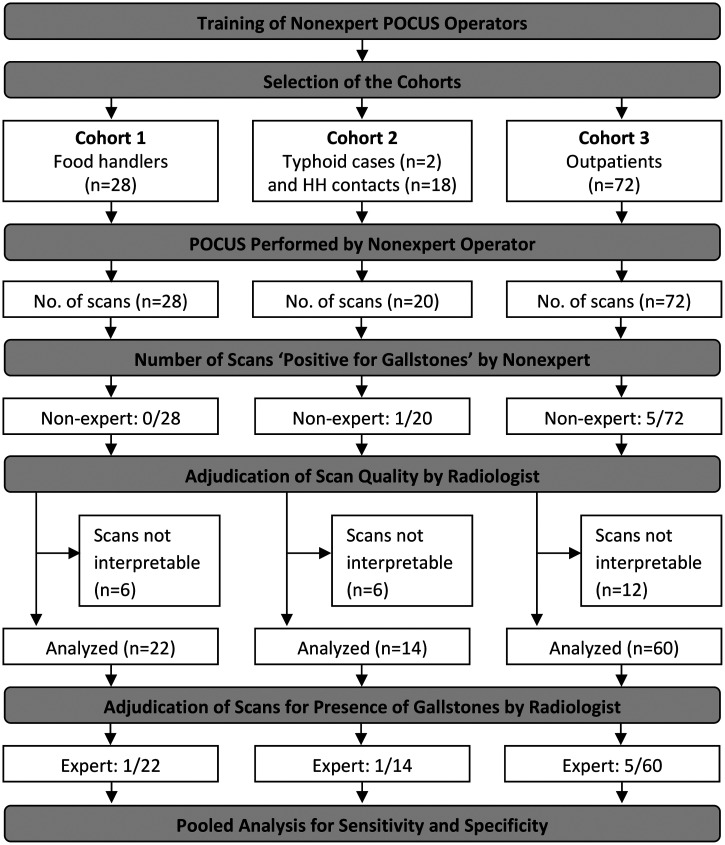
Summary diagram of enrolled participants and subsequent analysis. POCUS = point-of-care ultrasound; HH = household.

The workflow of this pilot evaluation of POCUS in the field to identify Samoan adults with gallstones is summarized in Figure 1. Of the 120 adults in the three cohorts who had POCUS scans performed by the two nonexpert operators, 24 (20%) were adjudicated by the expert radiologist as uninterpretable, leaving 96 interpretable scans. Scans were deemed uninterpretable primarily because of operator technique in failing to provide clear images of the gallbladder. The most common reason cited by the radiologist was “rapid operator movement” in which the nonexpert’s scan was performed at a speed too fast to provide high-quality images. The radiologist also noted that on some scans there were portions of the gallbladder where stones tend to reside (e.g., the neck of the gallbladder) that were not clearly profiled.

**Figure 2.**
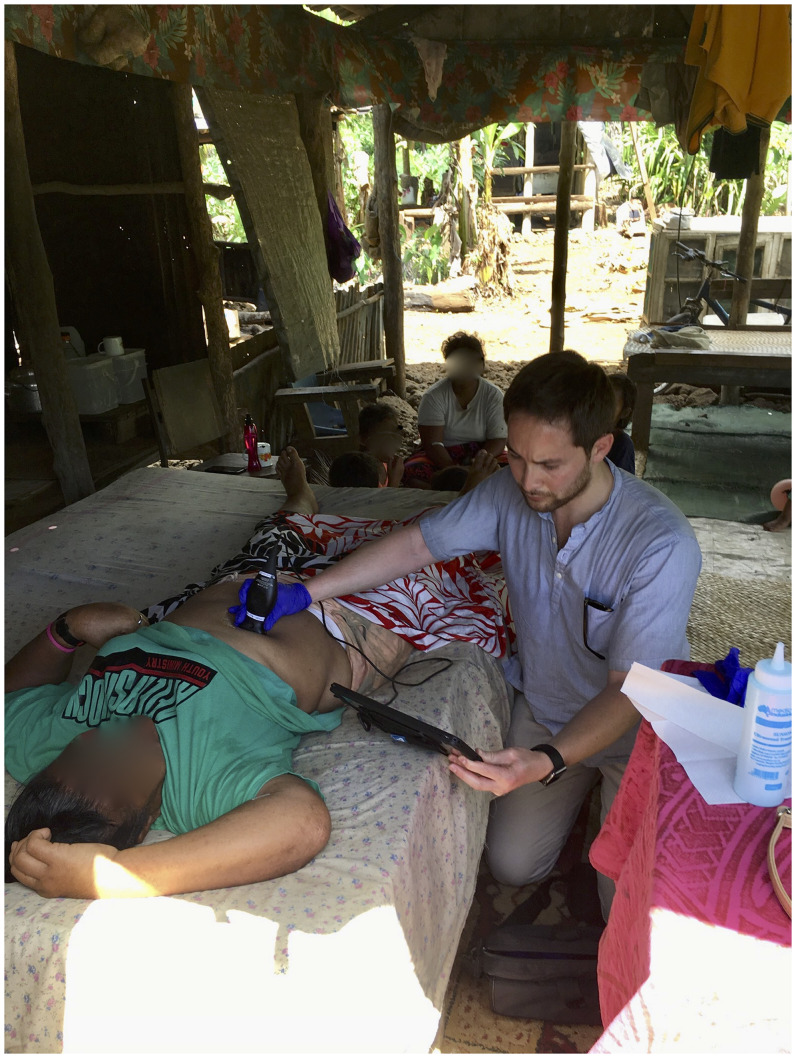
Nonexpert POCUS operator (S.A.H.) performing field examination for gallstones using the Butterfly iQ ultrasound probe. POCUS = point-of-care ultrasound.

**Figure 3.**
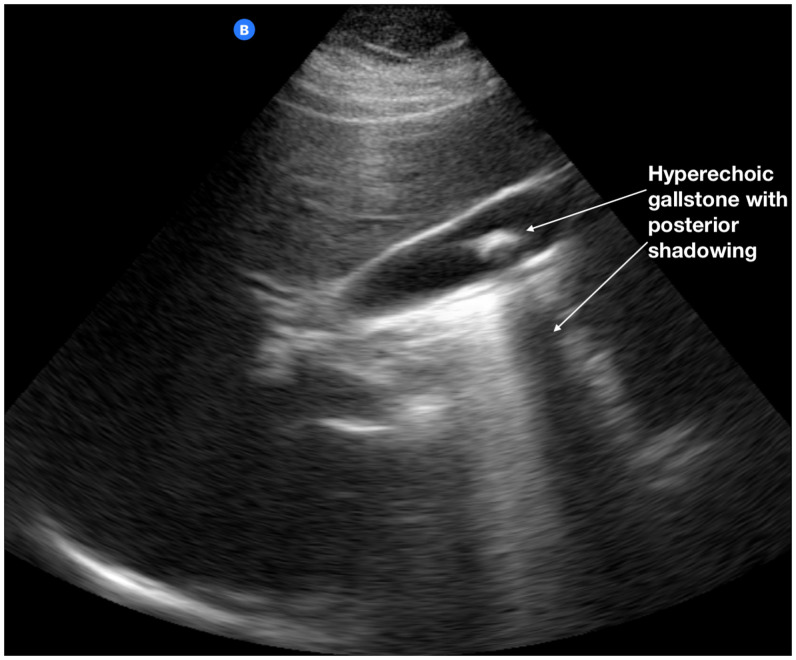
A single, large gallstone identified using the Butterfly iQ ultrasound probe in the course of screening performed by nonexpert POCUS operators. This POCUS image demonstrates the classical ultrasound characteristics of gallstones including a highly reflective echo from the anterior surface of the gallstone and marked posterior acoustic shadowing. With change of the subject’s position, the gallstone also typically changes position, which is readily detected during the POCUS examination. POCUS = point-of-care ultrasound.

The age group and sex of the 96 adults in the cohorts with interpretable scans and results of readings of the nonexperts are summarized in Table 1. Among the 96 (80%) scans of interpretable quality, the nonexperts correctly identified six of the seven Samoans that the radiologist diagnosed as having gallstones (Figure 3), thus exhibiting a sensitivity of 85.7% (95% CI, 42.1–99.6) (Table 2). The nonexperts correctly identified 85 of 89 scans that were determined by the radiologist to be negative for gallstones, yielding a specificity of 95.5% (95% CI, 88.9–98.8) (Table 2). A POCUS scan performed and interpreted by nonexperts had a positive predictive value that a subject truly has gallstones of 60% (95% CI, 35.5–80.4), and a negative predictive value that gallstones are truly not present of 98.8% (95% CI, 93.3–99.8).

**Table 1:** The prevalence of gallstones detected using a POCUS device among 96 of 120 Samoan women and men whose POCUS scans performed by nonexperts were deemed by a radiologist to be technically interpretable

All cohorts combined									
Age (yrs)	Females (n)	Gallstones (n)	% Positive	Males (n)	Gallstones (n)	% Positive	F + M (N)	Gallstones (N)	% Positive
20–39	9	1	11.1	11	0	0	20	1	5
40–59	23	3	13	20	1	5	43	4	9.3
60–79	16	0	0	15	2	13.3	31	2	6.5
≥ 80	1	0	0	1	0	0	2	0	0
Total	49	4	8.2	47	3	6.4	96	7	7.3
Food handlers (Cohort 1)									
20–39	4	1	25	9	0	0	13	1	7.7
40–59	4	0	0	4	0	0	8	0	0
60–79	1	0	0	0	0	0	1	0	0
≥ 80	0	0	0	0	0	0	0	0	0
Total	9	1	11.1	13	0	0	22	1	4.5
Acute typhoid cases and their household contacts (Cohort 2)									
20–39	3	0	0	1	0	0	4	0	0
40–59	3	1	33	2	0	0	5	1	20
60–79	3	0	0	2	0	0	5	0	0
≥ 80	0	0	0	0	0	0	0	0	0
Total	9	1	11.1	5	0	0	14	1	7.1
Among outpatients and accompanying persons (Cohort 3)									
20–39	2	0	0	1	0	0	3	0	0
40–59	16	2	12.5	14	1	7.1	30	3	10
60–79	12	0	0	13	2	15.4	25	2	8
≥ 80	1	0	0	1	0	0	2	0	0
Total	31	2	6.5	29	3	10.3	60	5	8.3

POCUS = point-of-care ultrasound

**Table 2:** Sensitivity and specificity of nonexpert versus expert diagnosis of gallstones using POCUS among 96 subjects with interpretable scans

		Presence of gallstones as confirmed by the board-certified radiologist	
		Positive	Negative
Presence of gallstones as detected by nonexpert POCUS operators	Positive	6^a^	4^b^
	Negative	1^c^	85^d^
Sensitivity and Specificity of nonexpert POCUS screen		Sensitivity: 85.7% (95% CI, 42.1–99.6%)	Specificity: 95.5% (95% CI, 88.9–98.8%)
		Sensitivity is defined as a/(a + c)	Specificity is defined as b/(b + d)

POCUS = point-of-care ultrasound

Among the 22 food handlers (Cohort 1) with scans deemed interpretable by the radiologist, one 37-year-old female had gallstones detected by the radiologist, but this woman’s scan was read as negative by the nonexperts; this individual was the one false negative.

Individuals in Cohort two with interpretable scans included two acute cases and 12 contacts. Neither of the typhoid fever patients, a 52-year-old female and a 31-year-old male, had gallstones. The 12 household contacts comprised eight females (30–72 years of age) and four males (45–79 years of age) (Table 1). One 49-year-old female was positive for gallstones; her three stool cultures did not grow *S*. Typhi. Thus, 1/14 (7.1%) acute cases of typhoid and their associated household contacts had gallstones.

Of the 60 patients, family members, and companions who availed themselves of the screening offered at routine outpatient health visits (Cohort 3) and who had scans deemed interpretable, 31 were females (20–80 years of age) and 29 were males (39–85 years of age) (Table 1). Among these, 2/31 (6.5%) females and 3/29 (10.3%) males had scans positive for gallstones (Table 1). Thus, overall, 5/60 (8.3%) of Cohorts 3 had gallstones.

## DISCUSSION

In our pilot study of the feasibility and applicability of portable, hand-held POCUS in the Samoan setting to detect gallstones among adults, nonexpert operators with minimal training were able to diagnose cholelithiasis with reasonably high sensitivity (85.7%) and high specificity (95.5%) among 96 scans of readable quality. These results closely mirror previously reported data on the ability of nonexpert operators to diagnose cholelithiaisis.^32^ The training of our nonexpert operators focused primarily on the diagnosis of cholelithiasis and less so on proper digital image recording for storage and future analysis. Additionally, the brief POCUS training did not include a large mixed population of female and male practice subjects with variable BMIs, and individuals known to harbor gallstones, sludge, or chronically inflamed biliary mucosa.

The percentage (20%) of scans deemed by a radiologist experienced in POCUS to be uninterpretable indicates that the nonexpert operators need additional training to improve their POCUS technique and mastery of the ultrasound software. Nevertheless, the fact that 80% of the POCUS scans performed by operators with minimal training were interpretable engenders optimism that with additional training the proportion of interpretable scans will increase. Perhaps more important is that in reviewing the interpretable scans the minimally trained clinicians did remarkably well in differentiating subjects with gallstones from those without.

To our knowledge this is the first report of the prevalence of gallstones in Samoans and in household contacts of typhoid fever patients in Samoa. However, this preliminary study is limited by a small sample size and a nonrandom approach to enrollment into the three cohorts, which together preclude analysis for demographic patterns or broad conclusions regarding the overall prevalence of gallstones in Samoa. Despite these limitations, the Ministry of Health has deemed the pilot to be sufficiently encouraging such that additional ultrasound probes have been acquired to create a cadre of Samoan clinicians who can undergo intensive training by radiologists and skilled technicians. We intend to undertake a future larger survey to assess the prowess of more highly trained Samoan POCUS operators. If a cadre of skilled POCUS operators gains sufficient proficiency, they will participate in Samoa Typhoid Epidemiologic SWAT Team activities. We envision that handheld POCUS will play a key role in active population-based screening of Samoans > 45 years of age to identify persons with cholelithiasis who will thereupon be further tested with stool cultures and Vi serology to detect chronic *S.* Typhi carriers, as part of the Consolidation Phase of the Samoa Typhoid Fever Control Program.
